# Growth and Spectral Assessment of Yb^3+^-Doped KBaGd(MoO_4_)_3_ Crystal: A Candidate for Ultrashort Pulse and Tunable Lasers

**DOI:** 10.1371/journal.pone.0054450

**Published:** 2013-01-22

**Authors:** Yi Yu, Yisheng Huang, Lizhen Zhang, Zhoubin Lin, Guofu Wang

**Affiliations:** 1 Key Laboratory of Optoelectronics Material Chemistry and Physics, Fujian Institute of Research on the Structure of Matter, Chinese Academy of Sciences, Fuzhou, Fujian, People’s Republic of China; 2 Graduate School of Chinese Academy of Sciences, Beijing, People’s Republic of China; University of Nottingham, United Kingdom

## Abstract

In order to explore new more powerful ultrashort pulse laser and tunable laser for diode-pumping, this paper reports the growth and spectral assessment of Yb^3+^-doped KBaGd(MoO_4_)_3_ crystal. An Yb^3+^:KBaGd(MoO_4_)_3_ crystal with dimensions of 50×40×9 mm^3^ was grown by the TSSG method from the K_2_Mo_2_O_7_ flux. The investigated spectral properties indicated that Yb^3+^:KBaGd(MoO_4_)_3_ crystal exhibits broad absorption and emission bands, except the large emission and gain cross-sections. This feature of the broad absorption and emission bands is not only suitable for the diode pumping, but also for the production of ultrashort pulses and tunability. Therefore, Yb^3+^:KBaGd(MoO_4_)_3_ crystal can be regarded as a candidate for the ultrashort pulse and tunable lasers.

## Introduction

With the development of high power InGaAs diode lasers, the Yb^3+^-doped laser materials have attracted great interest. The trivalent Yb^3+^ ion has only two electronic manifold, i.e. the ground state ^2^F_7/2_ and the excited state ^2^F_5/2_. The simple electronic-level scheme of Yb^3+^ ion can reduce the excited state absorption, quantum defect and concentration, which is helpful to improve the laser efficiency. In addition, the Yb^3+^ ion in the crystals exhibit strong and broad absorption and emission bands, which is beneficial to diode pumped ultrashort pulse lasers and tunable lasers. Recently, the laser crystals with disordered structure have been received much attention, because the disordered crystal structure can result in the broad absorption and emission bands of the laser crystals [1]–[5]. The powerful ultrashort pulse lasers have been achieved in some laser crystals, such as, Nd:SrGdGa_3_O_7_ [6] and Nd:CLNGG crystals [7]. Thus, when the Yb^3+^-ion doped into the crystal with disordered structure, can it further improve the spectral properties of Yb^3+^-doped laser crystal materials.

The KBaRe(MoO_4_)_3_ (Re = La-Lu, Y) compounds were first reported by N. M. Kozhevnikova et al [8]. The KBaGd(MoO_4_)_3_ crystal is a member of KBaRe(MoO_4_)_3_ (Re = La-Lu, Y) family. KBaGd(MoO_4_)_3_ crystal belongs to the monoclinic system with space group C2/c and cell unit parameters: a = 17.401 (11) Å, b = 12.226(8) Å, c = 5.324(4) Å, β = 106.19(1)°, V = 1087.73(373) Å^3^, Z = 4, D = 4.967 g.cm^−3^ [3]. Since the statistics of the structure have shown that the Ba and K ions in the crystal randomly occupy the same site with the same probability, KBaGd(MoO_4_)_3_ crystal has a high disordered structure [3]. This feature of structure could give further rise to the broad absorption and emission band of Yb^3+^ ion, which is beneficial to realize ultrashort pulse and tunable lasers. Here we report some preliminary results on Yb^3+^-doped KBaGd(MoO_4_)_3_ crystal.

## Materials and Methods

### 1. Crystal Growth

Since KBaGd(MoO_4_)_3_ crystal incongruently melts at 1054°C [3], it is only grown by the flux method. The 15 at.% Yb^3+^-doped KBaGd(MoO_4_)_3_ crystal was grown from a flux of K_2_Mo_2_O_3_ by the top solution seeding growth method (TSSG). The chemicals used were K_2_CO_3_, BaCO_3_ and MoO_3_ with purity 95%, La_2_O_3_ and Yb_2_O_3_ with purity of 99.99%. The starting materials consist of 17 mol% of solute (KBaGd(MoO_4_)_3_) and 83 mol% of solvent (K_2_Mo_2_O_3_). The weighed raw materials were mixed and put into a platinum crucible. Then, the full charged crucible was placed in vertical tubular furnace and slowly heated up to 1050°C, and kept this temperature for 2∼3 days to let the solution melt completely and homogeneously. Then a platinum wire was used as a seed to contact the solution, and the solution was slowly cooled down at a cooling rate of 15°C/day. The small crystals grown on the platinum wire were obtained by spontaneous crystallization. Then, a good small crystal was selected as a seed to grow the larger crystal. After exactly determining the saturation temperature by repeated seeding, the seed contacted the solution at a temperature 5°C above the saturation temperature for 30 min. The temperature was slowly cooled to 975°C to start growth. During the growth period, the crystal was slowly cooled at a cooling rate of 0.8∼1.5°C/day and rotated at a rotating rate of 15∼20 rpm. When the growth ended, the crystals were drawn out of the solution and cooled down to room temperature at a cooling rate of 15°C/h.

**Figure 1:**
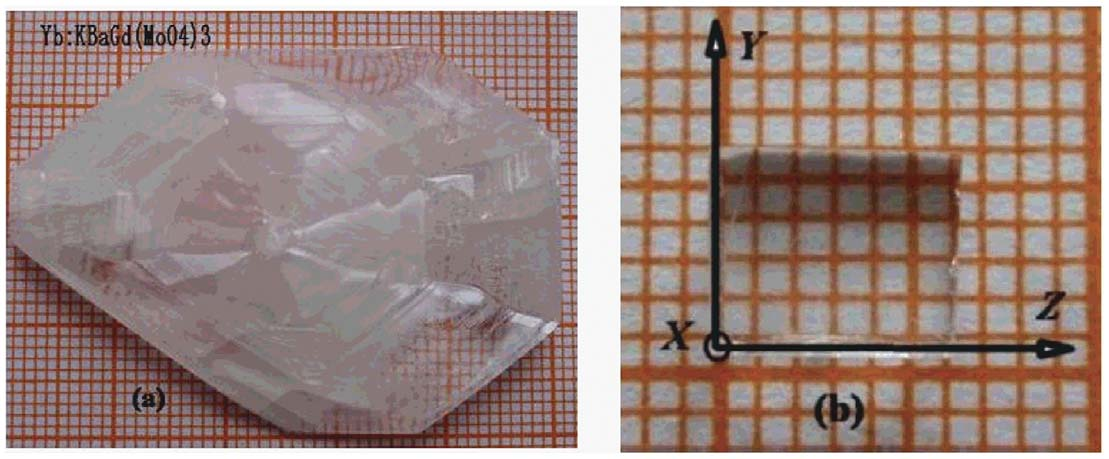
Picture of (a) Yb^3+^:KBaGd(MoO_4_)_3_ crystal, (b) polished sample cut from the crystal.

**Figure 2:**
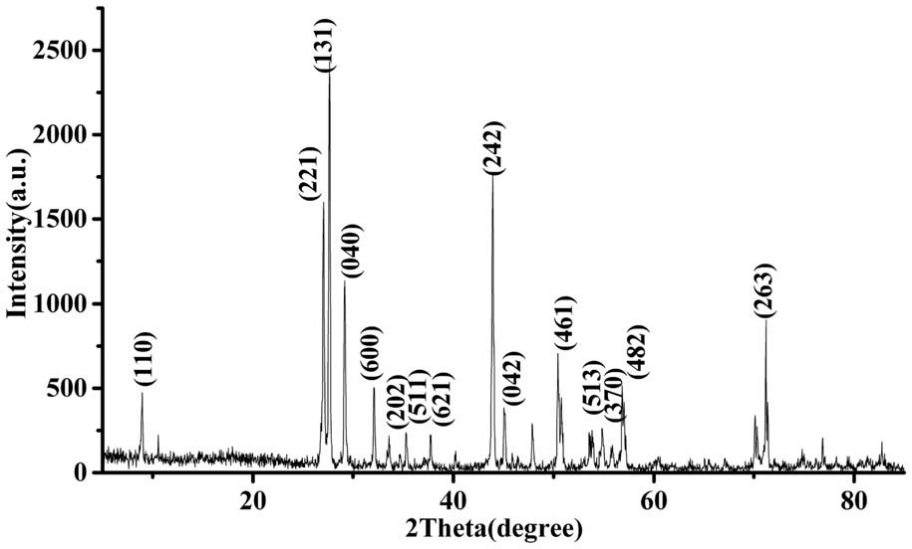
XRD pattern of Yb^3+^:KBaGd(MoO_4_)_3_ crystal at room temperature.

An Yb^3+^:KBaGd(MoO_4_)_3_ crystal with dimension of 50×40×9 mm^3^ was obtained, as shown in Fig. 1(a). The grown crystal was confirmed by the powder X-ray diffraction (XRD) using a CAD4 diffractometer equipped with CuKα radiation (λ = 1.054056Å). The XRD pattern of Yb^3+^:KBaGd(MoO_4_)_3_ crystal can be indexed according to the crystal structure of KBaGd(MoO_4_)_3_ crystal, as shown in Fig. 2, which confirmed that the grown crystal belongs to the Yb^3+^:KBaGd(MoO_4_)_3_ crystal The Yb^3+^ ion concentration in Yb^3+^:KBaGd(MoO_4_)_3_ crystal was measured to be 4.04 at.%, i. e. 1.494×10^20 ^cm^−3^ by inductively coupled plasma atomic emission spectrometry (ICP-AES).

### 2. Spectral Properties

**Figure 3:**
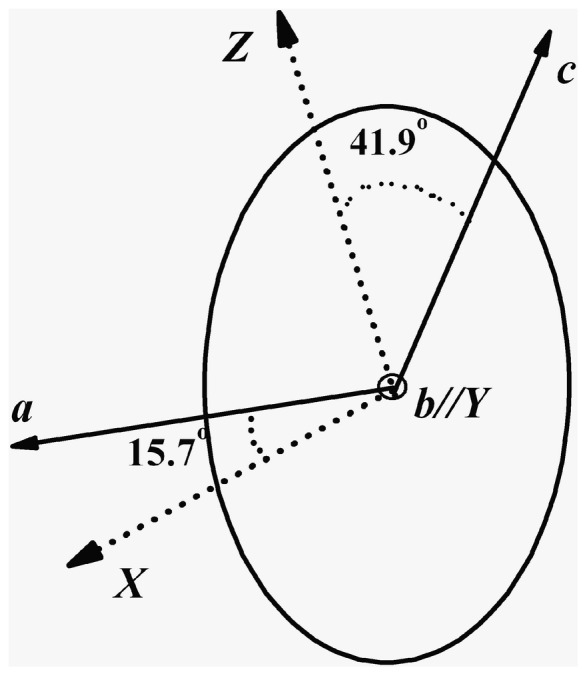
Orientation of principal axes *X*, *Y*, *Z* to the *a-,b-* and *c*-axis for Yb^3+^:KBaGd(MoO_4_)_3_ crystal.

Since Yb^3+^:KBaGd(MoO_4_)_3_ crystal belongs to monoclinic, the anisotropy of the crystal should be taken account. For the monoclinic crystal, the *Y* orthogonal principal crystallo-optic axe is parallel to the *b* Crystallography axe and the other two are in the *ac* plane. The orientation of the principal crystallo-optic axes *X*, *Z* to the *ac* axis was determined by using two crossed Glan–Taylor polarizer. Fig. 3 shows the sketch of the relationship between the optical axis and crystallography axis. A sample with dimension of 4.6×2.32×3.44 mm^3^ was cut from as-grown Yb^3+^:KBaGd(MoO_4_)_3_ crystal along the principal *X-*, *Y-* and *Z-* axes, as shown in Fig. 1(b). The sample was polished well and used for measuring the polarized absorption and fluorescence spectra at room temperature and low temperature. The polarized absorption spectrum was measured using a Perkin-Elmer UV-VIS-NIR spectrometer (Lambda-35) in a range of 900–1100 nm at room temperature. The polarized fluorescence spectra were recorded by a spectrophotometer (FLS920, Edinburgh) equipped with a xenon lamp as the excitation source. In the experiment the E-vector is parallel to the *X*-, *Y*- and *Z*-axis, respectively.

## Results and Discussion

### 1. Absorption Spectra

**Figure 4:**
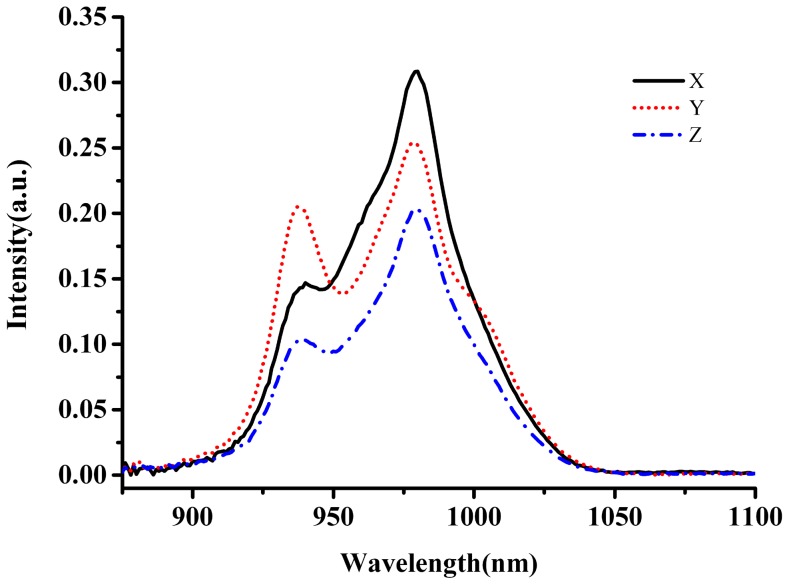
Polarized absorption of Yb^3+^:KBaGd(MoO_4_)_3_ crystal at room temperature.

**Figure 5:**
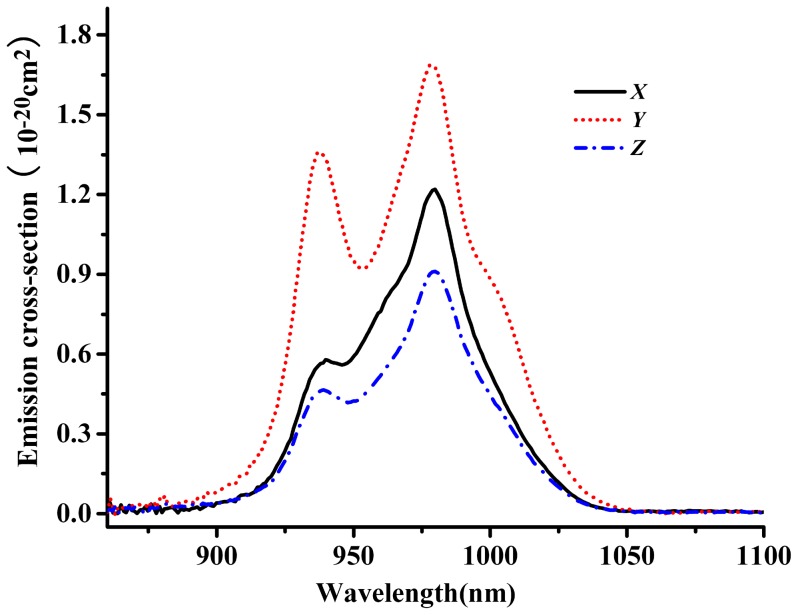
Polarized absorption cross-section of Yb^3+^:KBaGd(MoO_4_)_3_ crystal at room temperature.

The polarized absorption spectra of Yb^3+^:KBaGd(MoO_4_)_3_ crystal at room temperature is shown in Fig. 4, which exhibits a broad absorption feature. The absorption band has a very broad full-width at half-maximum (FWHM), which reaches to as higher as 45, 74 and 63 nm for the *X*-, *Y*- and *Z*-polarization at about 979 nm, respectively. In comparison with the other Yb^3+^-doped crystals (Table 1), the FWHM of Yb^3+^:KBaGd(MoO_4_)_3_ crystal is almost 10∼20 times broad than that of the other Yb^3+^-doped crystals. Such broad FWHM was further caused by the disordered structure of KBaGd(MoO_4_)_3_ crystal [3], except itself broad absorption and emission bands of Yb^3+^ ion. Since the output wavelength of diode laser is increased at 0.2∼.03 nm/°C with the operating temperature of the laser device, the temperature stability of the diode laser is needed to be crucially controlled. Therefore, such broad absorption band is very suitable for InGaAs diode laser-pumping. The absorption cross-sections were calculated based on the equation *σ_abs_ = α/N* where *N* is the Yb^3+^ ion concentration in Yb^3+^:KBaGd(MoO_4_)_3_ crystal, as shown in Fig. 5. The absorption cross-sections were calculated to be 1.22×10^−20^ cm^2^, 1.69×10^−20^ cm^2^ and 0.91×10^−20^ cm^2^ at 976 nm for the *X*-, *Y*- and *Z*-polarization, respectively.

**Table 1:** Spectral parameters of Yb^3+^:KBaGd(MoO_4_)_3_ and the other Yb^3+^-doped materials.

Spectral parameters	YAG	KY(WO_4_)_2_	KGd(WO_4_)_2_	Ca_4_GdO(BO_3_)_3_	Sr_3_Y(BO_3_)_3_	Ca_2_Nb_2_O_7_	KBaGd(MoO_4_)_3_		
							*X*	*Y*	*Z*
Absorption FWHM (nm) ∼at 976 nm	3	3.5	3.5	3	6	7.5	45	73	62
Zero-line wavelength (nm)	968	981	981	976	975	975	976.4	976.4	976.4
Pulse duration (fs)	340	71	112	89	69	–	–	–	–
Emission FWHM (nm)	10	16	20	44	60	57	59	81	67
Fluorescence lifetime (ms)	0.95	0.7	0.75	2.6	1.1	0.57	0.53		
σ_abs_ (10^−20^cm^2^)at zero-line	0.94	13.3	12	0.87	0.94	0.88	1.22	1.69	0.91
σ_em_ (10^−20^ cm^2^)	2.2	3	2.8	0.35	0.2	0.9	1.89	3.17	0.97
*I*_psat_ kW/cm^2^	27	53.67	9	25.5	20.5	40.8	60.9	44.1	81.6
*I*_min_ kW/cm^2^	1.4	3.94	0.8	1.54	1.31	3.1	10.5	7.6	14.1
*β*_min_	0.055	0.092	–	0.06	0.024	0.019		0.17	
Ref.	[13], [14]	[17], [20]	[18], [17], [20]	[9], [14], [19]	[14], [15], [19]	[21]	This work		

### 2. Fluorescence Lifetime

**Figure 6:**
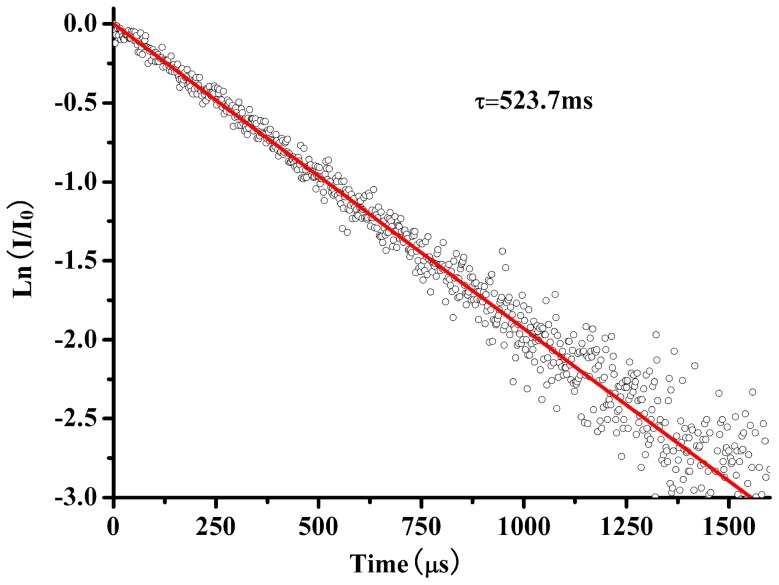
Lifetime decay curve of Yb^3+^:KBaGd(MoO_4_)_3_ crystal at room temperature.

**Figure 7:**
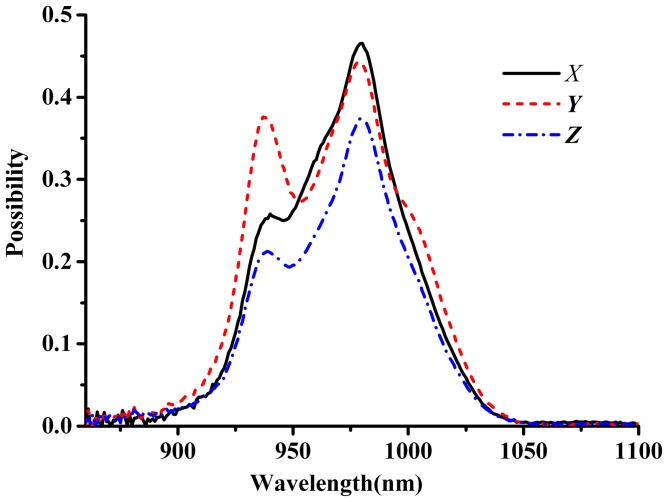
Re-absorption possibility for the three orientation of Yb^3+^:KBaGd(MoO_4_)_3_ crystal when *N_g_* = 1.494×10^20 ^cm^−3^, *l_X_ = l_Z_* = 0.344 cm and *l_Y_* = 0.232 cm.

**Figure 8:**
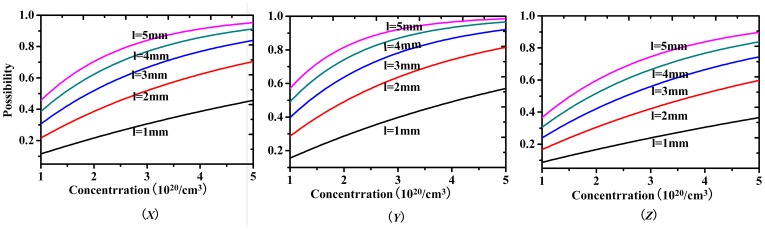
Relationship between the Yb^3+^ doping concentration and the re-absorption possibility at different absorption path length *l* when λ = 980 nm for the different polarizations in Yb^3+^:KBaGd(MoO_4_)_3_ crystal.

The radiative lifetime *τ_rad_* of Yb^3+^ ion in Yb^3+^:KBaGd(MoO_4_)_3_ crystal can be calculated Fortunately, it can be calculated from the absorption spectra by the follow formula [9]:
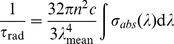
(1)where *λ_mean_* is the mean wavelength of the absorption peak (976 nm), *σ_abs_ (λ)* is the absorption cross-section at wavelength *λ*, *n* is reflective index which is 2.0 [3]. Thus, the radiative lifetime is calculated to be about 272.8 µs. The fluorescence lifetime *τ_f_* of the upper level was measured to be 523.7 µs, as shown in Fig. 6. The fluorescence lifetime is longer than the radiative lifetime, which is caused by re-absorption phenomenon, particularly in the circumstance of the bulk crystal. The re-absorption phenomenon reduces the possibility of photon transition from the ^2^F_5/2_ to the ^2^F_7/2_, so the fluorescence lifetime is longer than the real fluorescence lifetime of the ^2^F_5/2_ level. This calculated value is reliable when the re-absorption possibility is taken account. The re-absorption possibility of Yb^3+^ ion in Yb^3+^:KBaGd(MoO_4_)_3_ crystal can be examined by the following formula [10]

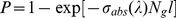
(2)where *P* is the re-absorption possibility, σ_abs_(λ) is the absorption cross-section at the same wavelength of the fluorescence photon. *Ng* is the concentration of Yb^3+^ ion in the ground state. The *l* represent the path length of fluorescence photo travels before it emits from the surface of the crystal sample, where *l_X_* = *l_Z_* = 0.344 cm and *l_Y_* = 0.232 cm, respectively. Fig. 7 shows the re-absorption possibility of the *X*-, *Y*- and *Z*-polarization in Yb^3+^:KBaGd(MoO_4_)_3_ crystal. From Fig. 7 it is easy to note all of the re-absorption possibilities in the three polarizations almost rise up to 0.5 at the wavelength of about 980 nm. This result proves that the calculated radiative lifetime, nearly half of the measured fluorescence lifetime, is reasonable. On the other hand, from formula (2), the path length of fluorescence photo traveled and the Yb^3+^-dopping concentration is also important factors to affect the re-absorption possibility. To investigate this effect, the re-absorption possibilities as the function of the Yb^3+^ ion concentration and path length are drawn in Fig. 8. When the absorption cross-section is fixed at the wavelength of 980 nm, the absorption cross-section is largest. Fig. 8 clearly gives the relationship between the Yb^3+^-doped concentration and path length the photon fluorescence travels. The re-absorption possibility increases dramatically with the path length rise up at the same Yb^3+^-doped concentration, especially in the higher concentration range. Similarly, in an anisotropic path length sample, the re-absorption possibility also changes a lot when the Yb^3+^-doped concentration propagates. For example, taking the *l* = 2 mm for the *Y*- polarization account, the possibility increase more than 3 times when the Yb^3+^-doped concentration rises form 1×10^20^ cm^−3^ to 5×10^20^ cm^−3^. Circumstances are almost the same for the *X*- and *Z*-polarizations.

### 3. Fluorescence Spectra

**Figure 9:**
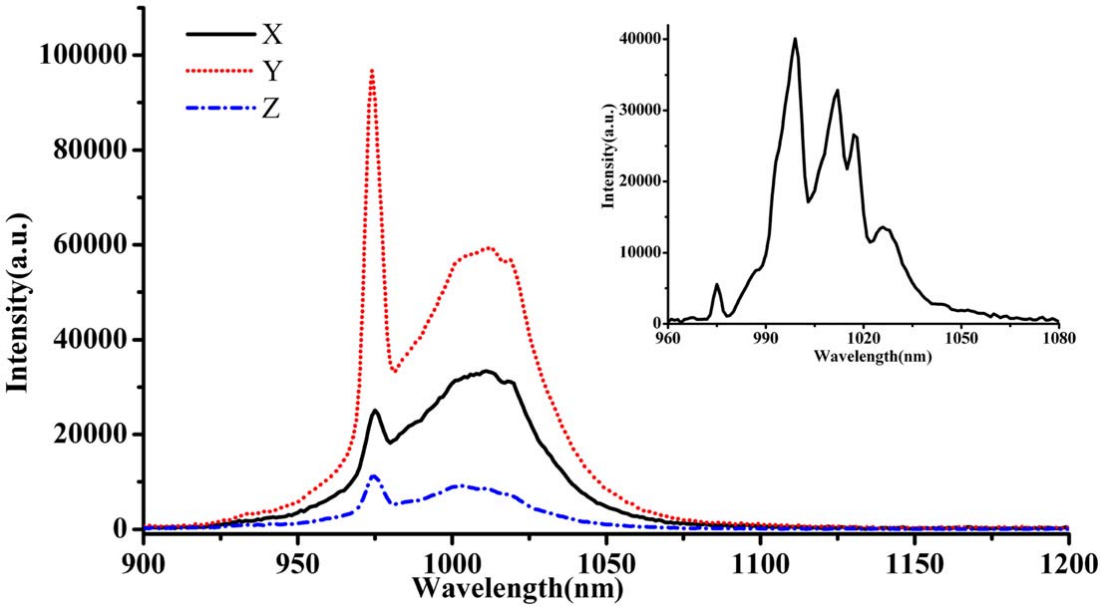
Polarized emission spectra of Yb^3+^:KBaGd(MoO_4_)_3_ crystal at room temperature and unpolarizied emission spectra at 10 K (insert).

**Figure 10:**
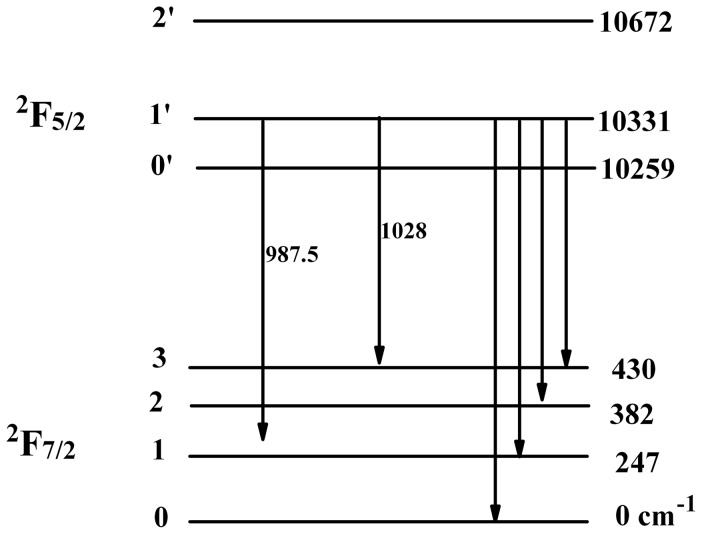
Stark energy-level diagram of the ^2^F_5/2_ and ^2^F_7/2_ manifold of Yb^3+^ in KBaGd(MoO_4_)_3_ crystal.

**Figure 11:**
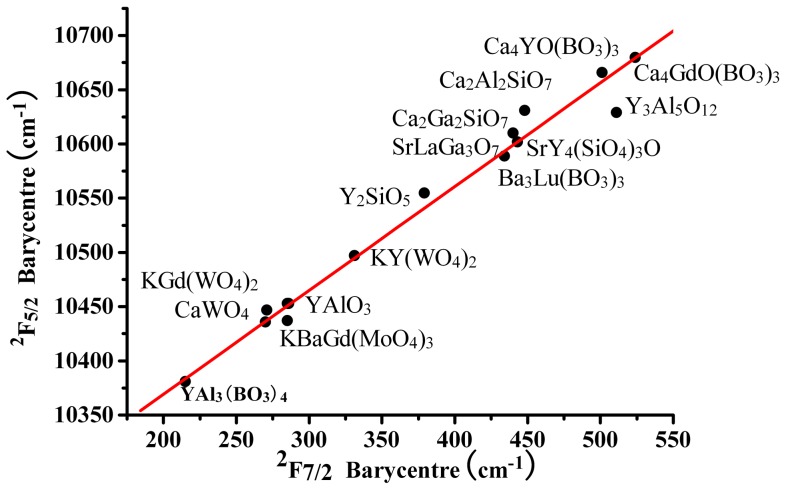
Barycentres plot for various Yb-doped materials.

The polarized emission spectra of Yb^3+^:KBaGd(MoO_4_)_3_ crystal at room temperature and un-polarized emission at 10 K are shown in Fig. 9. The emission spectra exhibited a broad emission bands. There is a sharp peak at about 976.4 nm in all of the polarized spectra, which is regarded as the zero-line. There are six peaks in the low temperature emission spectrum. Among them, four are corresponding to transitions from the lowest energy level of the ^2^F_5/2_ to the split^ 2^F_7/2_ level, and the other two could be signed to the transitions of the secondary lowest level of upper ^2^F_5/2_ to first and third levels of ^2^F_7/2_. Fig. 10 shows the energy levels of Yb^3+^:KBaGd(MoO_4_)_3_ crystal. To check the correction of identified stark energy-levels, a barycentres plot for various Yb^3+^-doped materials was presented in Fig. 11, including the Yb^3+^:KBaGd(MoO_4_)_3_ crystal [11], [12]. The dot representing Yb^3+^:KBaGd(MoO_4_)_3_ crystal appears very closes to the fitted line, which indicates that the identification stark energy-levels in Yb^3+^:KBaGd(MoO_4_)_3_ crystal is reliable.

The emission cross-section of ^2^F_5/2_→^2^F_7/2_ transitions of Yb^3+^:KBaGd(MoO_4_)_3_ crystal were usually calculate by the reciprocity method (RM) and Füchtbauer–Ladenburg method (F–L) [13]–[15]. It is reason that the RM method can only be employed if there is significant absorption, i. e. only in the vicinity of the fundamental transition. In other words, the RM method is not accurate at long wavelengths. The RM method is only suitable at short wavelength region. However, the F-L method is suitable for the long wavelength region because the re-absorption effect is not intense [10], [14]. Both methods are expressed as following:
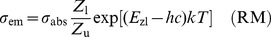
(3)

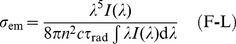
(4)


**Figure 12:**
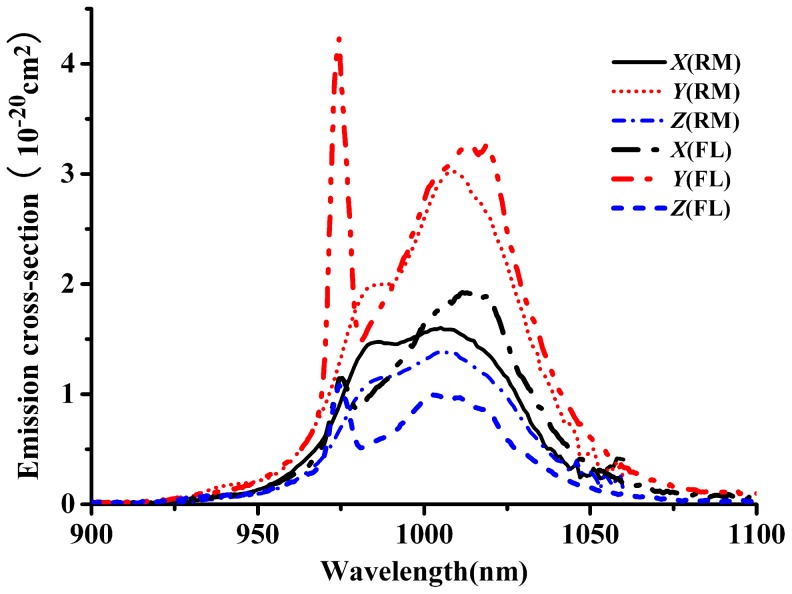
Emission cross-section of Yb^3+^ in KBaGd(MoO_4_)_3_ crystal calculated by the RM and F-L methods.

In the RM method, *Z*_l_ and *Zu* are partition functions for lower and upper levels, which can be calculated as follows:
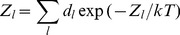
(5)

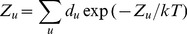
(6)
*k* is the Boltzmann’s constant, and *E*_ZL_ is the zero-line energy, which is defined as the energy separation between the lowest stark levels of ^2^F_5/2_ and ^2^F_7/2_ levels of Yb^3+^ ions. So based on the absorption and emission spectra, the zero line energy is confirmed to be at 974.8 nm and the Z_l_/Z_u_ is calculated to be 0.826. The emission cross-sections calculated by the two methods are shown in Fig. 12. Since above both methods are suitable for different range of wavelength, to calculate the emission FWHM needs to combine the results of both methods. In other words, the data of short wavelength is taken from the RM method and the data of long wavelength is taken from F-L method. Thus, Yb^3+^:KBaGd(MoO_4_)_3_ crystal has broad emission FWHM of 81 nm for *Y-*polarization. Again, the *Y* orientation exhibits larger emission cross-section than the other two orientations. Thus, the emission cross-sections at 1010 nm are 1.89, 3.17 and 0.97×10^−20^ cm^2^ for the *X-*, *Y-* and *Z-*polarization, respectively. In comparison with the other Yb^3+^-doped laser crystal materials (Table 1), Yb^3+^:KBaGd(MoO_4_)_3_ crystal has large emission cross-section and broad emission FWHM of 81 nm for *Y-*polarization. Such broad emission FWHM is caused by the disordered structure of Yb^3+^:KBaGd(MoO_4_)_3_ crystal, except itself broad emission of Yb^3+^ ion. As well known, the broadened emission band is the fundamental condition of realizing femtosecond laser. The broader the emission band is, the shorter laser pulse will be possible to obtain. Therefore, Yb^3+^:KBaGd(MoO_4_)_3_ crystal will be easier to achieve the output of ultra-short laser pulse than most reported Yb^3+^-doped crystals before.

### 4. Evaluation of Laser Potential

Based on the spectral parameters mentioned above, the important three laser performance parameters of *β*_min_, *I*_sat_ and *I*_min_ can be evaluated. The *β*_min_ represents the minimum inversion fraction of Yb^3+^ ions in the excited-state to achieve population inversion at the extraction wavelength. It was calculated by the following formula [16]:
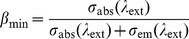
(7)


The minimum inversion fraction *β*_min_ of Yb^3+^ ions in Yb^3+^:KBaGd(MoO_4_)_3_ crystal was calculated to be 17.3% and 16.6% at 1010 nm for the RM and F-L methods, respectively.

The saturation pump intensity *I*_psat_, which is a measure of the ease of bleaching the material, can be determined by the following equation [16]:
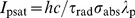
(8)


Then *I*_psat_ is calculated to be 60.9 KW/cm^2^, 44.1 KW/cm^2^and 81.65 KW/cm^2^ at 976 nm for the *X-*, *Y- and Z-* polarization, respectively. *I*_min_ is the minimum pump intensity to reach threshold at the extraction wavelength, which is important, too. The minimum pump intensity *I*_min_ was derived by
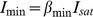
(9)


Then the minimum pump intensity *I*_min_ at the wavelength of 1010 nm were calculated to be 10.5 KW/cm^2^, 7.6 KW/cm^2^ and 14.1 KW/cm^2^ for the X-, Y- and Z-polarization, respectively.

The gain cross-section σ_g_ is another important parameter to evaluate the possible tuning range of laser wavelength and it can be derived form following equation:
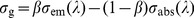
(10)


**Figure 13:**
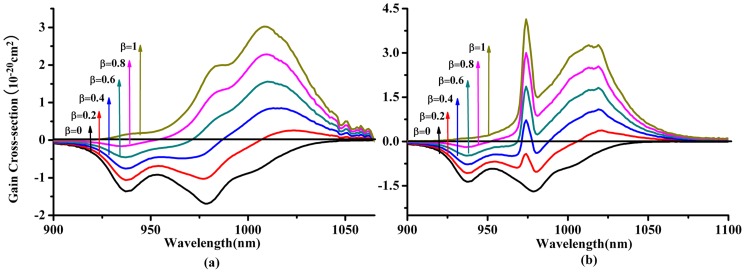
Gain profiles for the Y direction of Yb^3+^:KBaGd(MoO_4_)_3_ crystal obtained by two methods: (a) the RM method and (b) the F-L method.

Here *β* represents the excited state ions fraction. Since the Y-polarized emission spectrum has most broad and strong emission spectrum in Yb^3+^:KBaGd(MoO_4_)_3_ crystal, Fig. 13 gives the gain cross-section profiles for the *Y*-polarization. Yb^3+^:KBaGd(MoO_4_)_3_ crystal exhibits broad gain cross-sections. The result indicates broad wavelength tunabilty. The FWHM of gain band at β = 0.8 are 52 nm and 45 nm for the RM and F-L method, respectively.

### 5. Conclusion

A 4.04 at.% Yb^3+^:KBaGd(MoO_4_)_3_ crystal was grown by the TSSG method from the K_2_Mo_2_O_7_ flux. The Yb^3+^:KBaGd(MoO_4_)_3_ crystal has broad absorption and emission bands, except the large emission and gain cross-sections. This feature is not only suitable for the diode pumping, but also for the production of ultra-short pulses. Therefore, Yb^3+^:KBaGd(MoO_4_)_3_ crystal can be regarded as a candidate for the ulstrashort pulse and tunable lasers.
